# Pyruvate Dehydrogenase Kinase Inhibitor Dichloroacetate Improves Host Control of *Salmonella enterica* Serovar Typhimurium Infection in Human Macrophages

**DOI:** 10.3389/fimmu.2021.739938

**Published:** 2021-09-06

**Authors:** Cassandra L. R. van Doorn, Gina K. Schouten, Suzanne van Veen, Kimberley V. Walburg, Jeroen J. Esselink, Matthias T. Heemskerk, Frank Vrieling, Tom H. M. Ottenhoff

**Affiliations:** Department of Infectious Diseases, Leiden University Medical Center, Leiden, Netherlands

**Keywords:** host-directed therapy, dichloroacetate (DCA), pyruvate dehydrogenase kinase (PDK), Salmonella typhimurium, primary human macrophages

## Abstract

Global increases in the prevalence of antimicrobial resistance highlight the urgent need for novel strategies to combat infectious diseases. Recent studies suggest that host metabolic pathways play a key role in host control of intracellular bacterial pathogens. In this study we explored the potential of targeting host metabolic pathways for innovative host-directed therapy (HDT) against intracellular bacterial infections. Through gene expression profiling in human macrophages, pyruvate metabolism was identified as potential key pathway involved in *Salmonella enterica* serovar Typhimurium (*Stm*) infections. Next, the effect of targeting pyruvate dehydrogenase kinases (PDKs) – which are regulators of the metabolic checkpoint pyruvate dehydrogenase complex (PDC) – on macrophage function and bacterial control was studied. Chemical inhibition of PDKs by dichloroacetate (DCA) induced PDC activation and was accompanied with metabolic rewiring in classically activated macrophages (M1) but not in alternatively activated macrophages (M2), suggesting cell-type specific effects of dichloroacetate on host metabolism. Furthermore, DCA treatment had minor impact on cytokine and chemokine secretion on top of infection, but induced significant ROS production by M1 and M2. DCA markedly and rapidly reduced intracellular survival of *Stm*, but interestingly not *Mycobacterium tuberculosis*, in human macrophages in a host-directed manner. In conclusion, DCA represents a promising novel HDT compound targeting pyruvate metabolism for the treatment of *Stm* infections.

## Introduction

The rising prevalence of antimicrobial drug-resistance poses a serious threat to the control of bacterial infectious diseases. Drug-resistance among common bacterial infections continues to cause an estimated mortality rate of 700,000 people annually (1). Two major examples of antibiotic-resistant pathogens are *Mycobacterium tuberculosis* (*Mtb*) and *Salmonella enterica*. The World Health Organization estimated that around 10 million people developed active tuberculosis (TB) in 2017, and that 3.5% of new TB cases and 18% of previously treated TB cases were infected by rifampicin-resistant *Mtb* strains or multidrug-resistant *Mtb*-strains (2). *S. enterica* is a common food-borne pathogen that can cause gastroenteritis and typhoid fever, the latter being responsible for an estimated 200,000 human deaths annually (3). Non-typhoidal *S. enterica* caused invasive disease in 535,000 people in 2017 and two highly invasive drug-resistant *S. enterica* serovar Typhimurium (*Stm*) strains have been described (4–6). Drug-resistance in intracellular pathogens such as *Mtb* and *Stm* is a major concern to global health and poses a critical need for developing new and more effective treatments against such bacteria.

Host-directed therapy (HDT) aims at augmenting host immune responses, mostly as adjunct therapy to currently used antibiotics. HDT may not only provide more effective treatment regimens but may also help shortening current lengthy treatment regimens and thus reducing risk of de-novo resistance mutations as well as reducing antibiotics-induced adverse effects (7). The development of novel HDT, however, requires a better understanding of the biology of intracellular infections and bacterial survival tactics. Recent studies have connected regulation of host defense against bacteria with host metabolic pathways (8). Rapid upregulation of glycolysis in macrophages is a hallmark of *Mtb* infection and LPS stimulation, and resembles the Warburg effect seen in cancer cells, but the mechanisms underlying this metabolic remodeling in bacterially-infected host cells remain poorly understood. Although multiple studies have suggested that rapid upregulation of glycolysis favors host immunity by meeting urgent energy demands required to induce protective immune responses (9–16), more recent studies have shown that tricarboxylic acid (TCA) cycle intermediates, including itaconate, citrate and succinate, are associated with host immune responses against intracellular pathogens as well (17–24). Host metabolic pathways and immune control of intracellular pathogens are thus interconnected in a highly complex manner. The specific immunometabolic pathways underlying macrophage-mediated inflammation and bacterial control remain largely unresolved. This particularly applies to infection-induced metabolic remodeling of human macrophages, which is significantly underrepresented in the literature. In the current study, we therefore modelled host-pathogen interactions in two different, polar human macrophage subsets which are known to respond differently to external stimuli. Classically activated macrophages (M1) display a pro-inflammatory phenotype and function in response to pathogen-associated molecular patterns (PAMPS) whereas alternatively activated macrophages (M2) display an anti-inflammatory phenotypes and function in response to PAMPS (25, 26). Although the metabolic divergence between M1 and M2 is well described for murine macrophages, surprisingly little is known about human monocyte-derived macrophages (27–29).

Here, we identify the metabolic impact of infections with intracellular bacteria *Stm* and *Mtb* on primary human macrophages and report the prominent effect of the metabolic checkpoint pyruvate dehydrogenase complex (PDC) on *Stm* but not *Mtb* infections. PDC catalyzes the conversion of pyruvate into acetyl-CoA to supply the TCA cycle and is a gate-keeping enzyme regulating the balance between glycolysis and oxidative phosphorylation (OXPHOS). PDC consists of three subunits: pyruvate dehydrogenase (PDH, subunit E1), dihydrolipoamide acetyltransferase (subunit E2) and dihydrolipoamide dehydrogenase (subunit E3). PDH activity is the rate-limiting step during the conversion of pyruvate into acetyl-CoA (30). PDH activity is blocked upon phosphorylation by pyruvate dehydrogenase kinases (PDKs) and restored upon dephosphorylation by pyruvate dehydrogenase phosphatases (PDPs) (31). PDK inhibitors have been developed for treating cancer and type II diabetes (32–34).

In the current study, we report that PDKs are significantly modulated during *Stm* infection in primary human macrophages at the transcriptional level. Dichloroacetate (DCA), a chemical inhibitor of PDKs, activated PDC in M1 and M2 and induced a metabolic shift from glycolysis to OXPHOS in M1. DCA markedly increased production of reactive oxygen species (ROS) by M1 and M2. DCA induced a significant reduction of intracellular *Stm* (but not *Mtb*) in human macrophages, but did not exert a direct antibiotic effect on *Stm* extracellular bacteria, indicating that DCA induces *Stm* control *via* modulation of host metabolic pathways. In conclusion, DCA represents a promising novel HDT compound targeting pyruvate metabolism for the treatment of difficult to treat *Stm* infections.

## Results

### Differential Impact of *Stm Versus Mtb* Infection-Induced Glycolysis in Primary Human Macrophages

**Figure 1:**
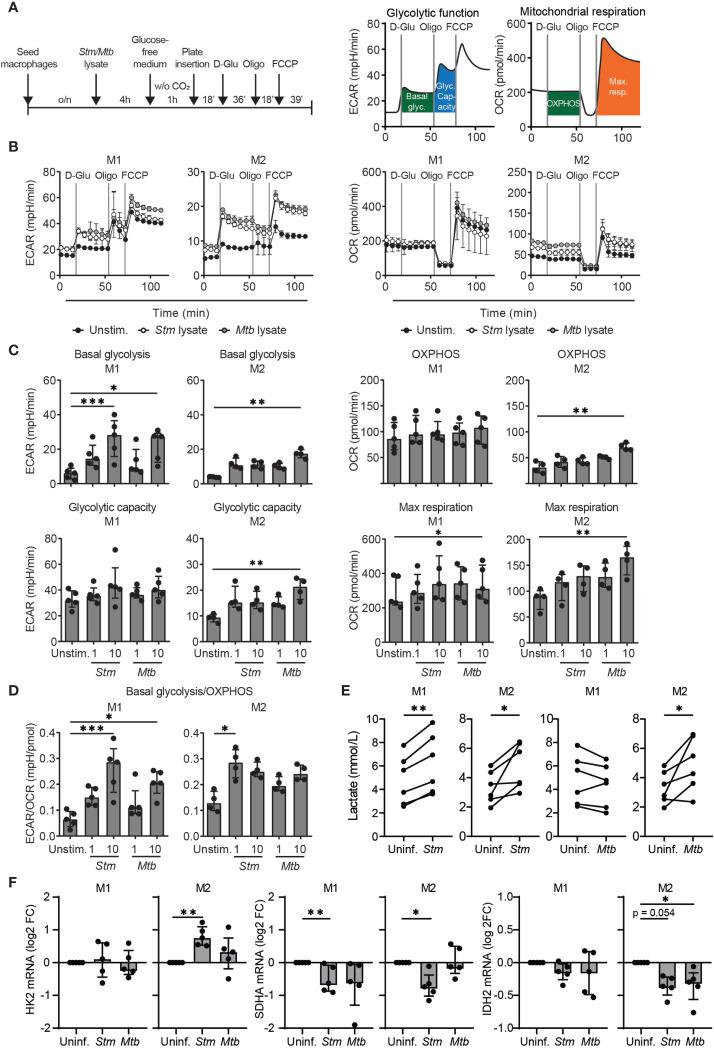
*Stm* and *Mtb* induced a more glycolytic phenotype in human macrophages. **(A)** Schematic representation of the experimental setup used in **(B–D)** (left panel) and a representative ECAR and OCR profile indicating basal glycolysis (basal glyc.), glycolytic capacity (glyc. capacity), OXPHOS and maximal respiration (max. resp.) (right panel). M1 and M2 were unstimulated (unstim.) or stimulated with 1 µg/ml *Stm* or *Mtb* lysate for 4 hours and inserted in the Seahorse analyzer. D-glucose (10 mM), oligomycin (oligo, 1 µM) and carbonyl cyanide 4-(trifluoromethoxy)phenylhydrazone (FCCP, 2 µM) were sequentially injected after 18, 54 and 72 minutes, respectively. **(B)** ECAR (mpH/min, left panel) and OCR (pmol/min, right panel) profiles of one representative donor out of five (M1) or four (M2) donors analyzed. Data represent the mean ± S.D. of triplicates. **(C, D)** Basal glycolysis, OXPHOS, glycolytic capacity, maximum respiration and the ratio between basal glycolysis and OXPHOS (ECAR/OCR) in M1 (left panels) and M2 (right panels). Data represent the median ± interquartile range of minimally four donors. Differences were significant by RM one-way ANOVA with Dunnett’s multiple test correction against the unstimulated control. **(E)** Lactate production was measured in the supernatant of M1 and M2 that were infected with *Stm* or *Mtb* overnight. Lines connect data points from the same donor, with a total of six donors tested. Significant differences were tested using a paired t-test. **(F)** HK2, SDHA and IDH2 mRNA expression levels were quantified in *Stm*- or *Mtb*-infected M1 and M2 at 4 hours post infection using GAPDH as the housekeeping control gene. Data represent the median ± interquartile range of five donors. Differences were significant by RM one-way ANOVA with Dunnett’s multiple test correction against the uninfected control. *p < 0.05, **p < 0.01, ***p < 0.001.

Although LPS stimulation and bacterial infections have been shown to shift cellular metabolism towards a more glycolytic phenotype in various cell types (9–16), a number of studies have reported that human macrophages do not increase glycolysis in response to bacterial stimuli (35, 36). To assess the potential of targeting host metabolic pathways to treat intracellular bacterial infections through HDT, we evaluated the effect of *Stm* and *Mtb* on cellular metabolism in primary human macrophages. Extracellular acidification rates (ECAR) and oxygen consumption rates (OCR) were measured as indicators of glycolytic and OXPHOS energy phenotypes, respectively, using a Seahorse XFe96 Extracellular Flux Analyzer(experimental outline in **Figure 1A**). Macrophages were pretreated with bacterial lysates, which we used as a proxy for live infections, due to safety restrictions in the Seahorse XFe96 Extracellular Flux Analyzer laboratory. At baseline, M1 displayed a higher general metabolic activity compared to M2 as evidenced by elevated basal glycolysis and OXPHOS levels (**Figures 1B, C**; note the different scales on the y-axes**)**. Basal glycolysis was increased in a dose-dependent manner upon 4 hours stimulation with 1 or 10 µg/ml *Stm* or *Mtb* lysate, both in M1 and in M2 compared to unstimulated macrophages (**Figures 1B, C** and **Supplemental Figure 1**). OXPHOS was significantly increased upon stimulation with 10 µg/ml *Mtb* lysate in M2, but not upon stimulation with *Stm* lysate. The maximum respiration and glycolytic capacity were significantly increased upon stimulation with 10 µg/ml *Mtb* lysate in M1 and M2 or M2 only, respectively, but not upon stimulation with *Stm* lysate or a low concentration (1 µg/ml) of *Mtb* lysate. Calculation of the ECAR/OCR ratios to determine the overall metabolic shift induced in human M1 and M2 following stimulation with *Stm* or *Mtb* demonstrated a bacterially-induced shift towards a more glycolytic phenotype (**Figure 1D**).

To assess modulation of metabolism by live bacteria, extracellular lactate levels (as an indicator of glycolysis) were determined in macrophages infected with live *Stm* and *Mtb* (**Figure 1E**). M1 and M2 secreted significantly higher levels of lactate upon *Stm* infection compared to uninfected cells, supporting infection-induced glycolysis. Live *Mtb* infection significantly increased lactate production in M2 compared to uninfected cells, but surprisingly not in M1. The absence of lactate production by *Mtb*-infected M1 corroborates recent findings in mouse bone marrow-derived macrophages (BMDMs) and human monocyte-derived macrophages that *Mtb* is able to actively suppress induction of glycolysis (37, 38).

To confirm induction of a metabolic shift at the transcriptional level, we studied mRNA expression levels of the glycolytic key gene hexokinase 2 (*HK2*) and the TCA cycle key genes succinate dehydrogenase complex flavoprotein subunit A (*SDHA*) and isocitrate dehydrogenase 2 (*IDH2*) in M1 and M2. *HK2* mRNA levels were significantly increased 4 hours post *Stm* infection in M2 (**Figure 1F**), but were not altered in *Stm*- or *Mtb*-infected M1(**Figure 1B**). *SDHA* and *IDH2* mRNA levels were downregulated in *Stm*- and *Mtb*-infected M1 and M2, albeit to different extents, suggesting decreased activity of the TCA cycle pathway. Taken together, our data suggest that *Stm* infections are able to shift human M1 and M2 metabolism towards a more glycolytic phenotype.

**Figure 2:**
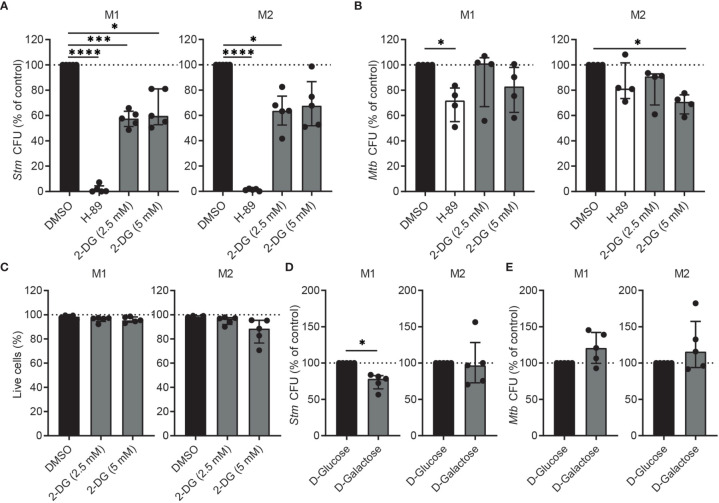
Inhibition of glycolysis decreased intracellular *Stm* and tended to increase intracellular *Mtb* in human macrophages. **(A, B)** *Stm* and *Mtb* CFUs in M1 (left panels) or M2 (right panels) after treatment with H-89 (10 µM), 2-DG (2.5 or 5 mM) or vehicle control DMSO (0.1% v/v) overnight. Data represent the median ± interquartile range of minimally four donors. CFUs are expressed as percentage of DMSO. Differences were significant by RM one-way ANOVA with Dunnett’s multiple test correction against DMSO. **(C)** Percentage of live cells (i.e. Hoechst-positive, PI-negative cells) after overnight treatment with 2-DG (2.5 or 5 mM) or vehicle control DMSO (0.1% v/v) in M1 (left panel) or M2 (right panel). Data represent the median ± interquartile range of five donors. Differences were tested by RM one-way ANOVA with Dunnett’s multiple test correction against DMSO. **(D, E)** *Stm* and *Mtb* CFUs in M1 (left panels) or M2 (right panels) cultured in medium containing D-glucose (11 mM) or D-galactose (11 mM) for 24 hours. Data represent the median ± interquartile range of five donors. CFUs are expressed as percentage of DMSO. Significant differences were tested using a paired t-test. *p < 0.05, ***p < 0.001, ****p < 0.001.

To study whether the increased glycolysis contributes to host defense, we first treated *Stm*- and *Mtb*-infected M1 and M2 with glycolysis inhibitor 2-deoxy-D-glucose (2-DG), since earlier reports showed that 2-DG increased the burden of *Mtb* in murine BMDMs and in mouse lungs (39, 40). PKA inhibitor H-89 was used as positive control and in agreement with previous findings (41, 42) significantly lowered outgrowth of *Stm*, while being less effective in reducing *Mtb* bacterial load (**Figures 2A, B****)**. Surprisingly, treatment of human M1 with 2-DG significantly decreased outgrowth of intracellular *Stm*, but only decreased intracellular *Mtb* in M2 at the highest concentration (5 mM). Importantly, 2-DG did not show cytotoxicity in both macrophage subsets (**Figure 2C**). The finding that 2-DG treatment did not (M1) or only moderately (M2) affect *Mtb* bacterial load in primary human macrophages contrasts with earlier reports, which concluded that 2-DG treatment increased the burden of *Mtb* in murine BMDMs and in mouse lungs (39, 40). However, 2-DG has recently been described to decrease *Legionella pneumophila* survival independently of inhibition of glycolysis in mouse BMDMs (43) and also, high concentrations of 2-DG have been shown to inhibit not only glycolysis but also OXPHOS in mouse BMDMs (44). Therefore, in order to be able to evaluate the effect of glycolysis independently from the use of 2-DG, we determined bacterial survival after substitution of D-glucose by D-galactose in the cell culture medium. Substitution of D-glucose by D-galactose reduces glycolytic flux and thus could independently validate our findings (44). Indeed, the absence of glucose significantly decreased *Stm* outgrowth in M1, but not M2, and tended to increase intracellular *Mtb* levels (**Figures 2D, E****)**. These data suggest that inhibition of glycolysis impacts *Stm* and *Mtb* in opposite directions; it can inhibit *Stm* outgrowth, while it can promote *Mtb* survival as described (39, 40). Also of importance, these data suggest that pathways other than inhibition of glycolysis are responsible for the antibacterial effect of 2-DG.

### Gene Expression Analysis Identifies Pyruvate Dehydrogenase Kinases as Potential Host Targets for HDT During *Stm* and *Mtb* Infections

Next, we aimed to identify metabolic key genes that are modulated during *Stm* and *Mtb* macrophage infections. We performed computational analysis of published RNAseq data by Blischak et al. (45) to study the expression of metabolic genes (i.e. all genes in Reactome pathways ‘Glucose metabolism’ and ‘TCA cycle and respiratory electron transport’) in human M2 macrophages infected for 18 hours with *Stm* or *Mtb* (MOI of 5) compared to their time-matched controls (mock infection). *Stm* infection had a more prominent effect on the regulation of glycolytic genes and genes involved in the TCA cycle and respiratory electron transport compared to *Mtb* (**Supplemental Figure 2**). Two key glycolytic genes, enolase (*ENO*) 2 and *HK2*, were significantly upregulated 18 hours post *Stm* or *Mtb* infection. In contrast, most genes involved in the TCA cycle and respiratory electron transport were downregulated upon *Stm* and *Mtb* infection.

**Figure 3:**
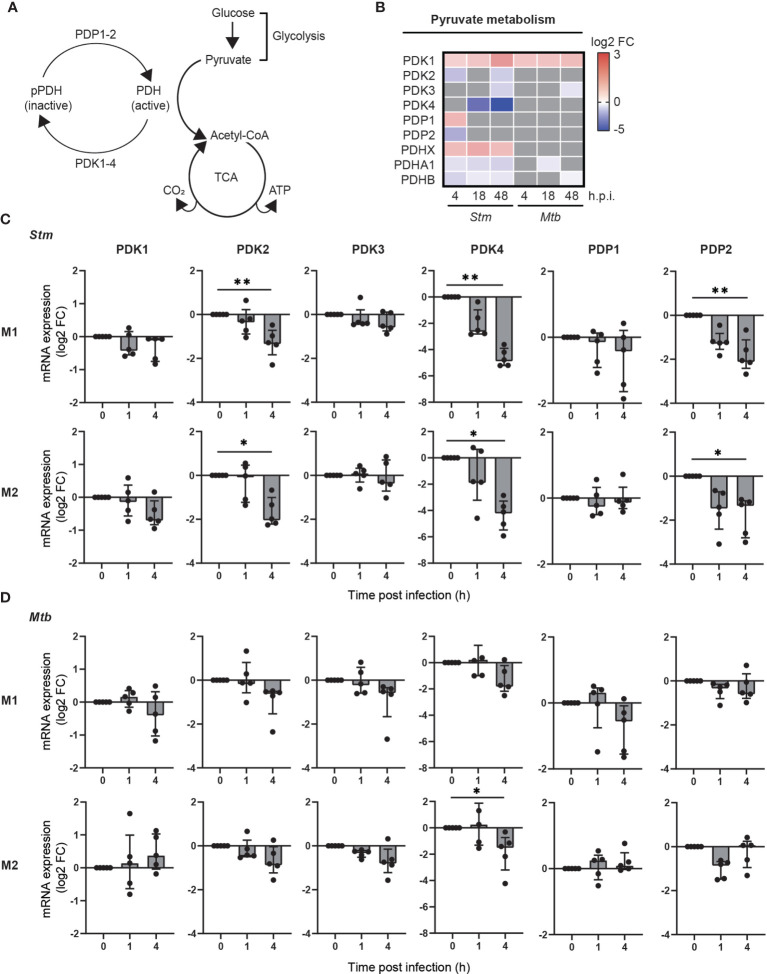
Pyruvate metabolism was regulated during Stm infection at the transcriptional level in human macrophages. **(A)** Schematic representation of the PDC, linking glucose metabolism with the TCA cycle and respiratory electron transport. **(B)** Differential expression analysis by limma-voom of genes involved in pyruvate metabolism over time in *Stm*- or *Mtb*-infected relative to uninfected M2 macrophages. Significantly up- (red) or downregulated genes (blue) (adjusted p-value < 0.05) are indicated on a log_2_-FC scale. Grey indicates non-significantly differentially expressed genes. Data obtained from Blischak et al. (45) **(C, D)** mRNA expression levels were quantified in *Stm*- or *Mtb*-infected M1 (top panels) and M2 (bottom panels) prior to infection (0) or 1 or 4 hours post infection using GAPDH as the housekeeping control gene. Data represent the median ± interquartile range of five donors. Differences were significant by RM one-way ANOVA with Dunnett’s multiple test correction against the corresponding uninfected control. *p < 0.05, **p < 0.01.

We hypothesized that metabolic genes that are modulated during *Stm* and *Mtb* infections may provide novel targets for HDT. Surprisingly, among the most up- and downregulated metabolic genes during *Stm* and *Mtb* infection, two PDK isoforms were regulated in opposite directions: *PDK4* was strongly downregulated upon *Stm* infection, whereas *PDK1* was strongly upregulated upon *Mtb* infection (**Supplemental Figure 3**). The differential expression of PDK isoforms is interesting, because PDKs are regulators of the metabolic checkpoint PDC, linking glycolysis with TCA cycle activity and OXPHOS (**Figure 3A**). More detailed kinetic analysis of the expression levels of all PDK isoforms (*PDK1-4*) and other components of the PDC (*PDP1, PDP2, PDHX, PDHA1 and PDHB*) showed that several PDK isoforms and catalytic subunits of the PDC were significantly regulated upon infection with *Stm*, in contrast to infection with *Mtb* (**Figure 3B**), suggesting that pyruvate metabolism was modulated in human macrophages during *Stm* infection.

To confirm and extend the data by Blischak et al. to both M1 and M2 infection models, we measured the expression changes of genes involved in activation of the PDC (i.e.: *PDK1-4, PDP1-2*) upon infection with *Stm* and *Mtb* in both M1 and M2. In agreement with the data obtained by Blischak et al., gene expression levels of *PDK2, PDK4* and *PDP2* were significantly downregulated in *Stm*-infected human M1 and M2 (**Figure 3C**). A similar trend could be observed in *Mtb*-infected macrophage subsets, albeit not significant (**Figure 3D**). Taken together, these data indicate that pyruvate metabolism is markedly modulated during *Stm* infections and to a lesser extent during *Mtb* infections in human M1 and M2, as shown by a downregulation of *PDK2*, *PDK4* and *PDP2* at the transcriptional level.

### Dichloroacetate Can Be Used as a Tool to Study the Effect of Pyruvate Dehydrogenase Kinases on Intracellular *Stm* and *Mtb* Control in Human Macrophages

**Figure 4:**
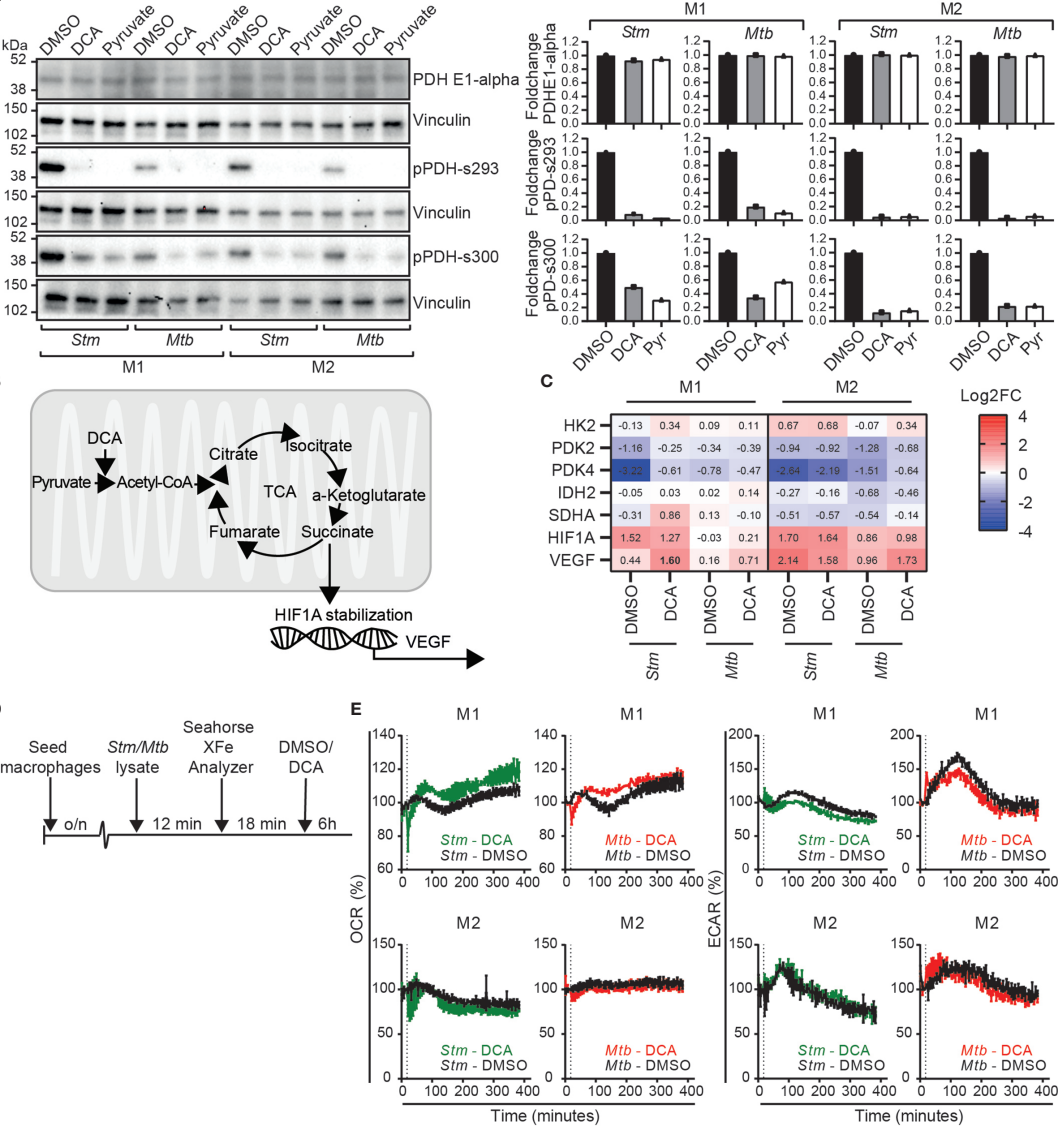
DCA activated the PDC in human macrophages and induced a metabolic shift from glycolysis to OXPHOS in M1. **(A)** Western blot showing total PDH (PDH-E1α) and PDH phosphorylation at serine residues 293 and 300 at 4 hours post infection in *Stm-* or *Mtb*-infected M1 and M2 treated with DCA (10 mM), pyruvate (10 mM) or vehicle control DMSO (0.1% v/v). Shown is a representative blot from one out of three independent experiments (left panel) and the band intensities were quantified using Fiji/ImageJ, normalized to loading control vinculin and shown as fold change (FC) against DMSO (right panel). **(B)** Schematic representation of pyruvate metabolism linked with the TCA cycle and HIF-1α stabilization. **(C)** mRNA expression levels were quantified by Fluidigm qPCR using GAPDH as the housekeeping control gene. *Stm*- or *Mtb*-infected M1 and M2 were treated for 4 hours with DCA (10 mM) or vehicle control DMSO (0.1% v/v). Shown are the median ± interquartile range log2FC relative to uninfected macrophages of four donors. Differences were significant by a paired t-test between DMSO and DCA and are shown in bold. **(D)** Schematic representation of the experimental setup used in **(E)**. **(E)** The ECAR and OCR were determined over time (0-400 min) in *Stm* or *Mtb*-lysate stimulated human macrophages using the Seahorse XF96 analyzer. M1 and M2 were stimulated with 10 µg/ml *Stm* or *Mtb* lysate for approximately 12 minutes before the first measurement. The vertical dashed line indicates DCA (final concentration 10 mM) or DMSO (at equal v/v) injection at t=18 minutes. Data represent the mean ± standard deviation of triplicates from one representative donor out of four donors.

To study the effect of pyruvate metabolism during *Stm* and *Mtb* infections in more detail, we aimed to modulate this pathway through chemical inhibition of PDK using dichloroacetate (DCA). To validate inhibition of PDKs as the mechanistic target of DCA, we first examined whether DCA treatment in *Stm* and *Mtb*-infected macrophage subsets resulted in dephosphorylation of PDH at serine residues 293 and 300, which corresponds with increased PDC activation. Pyruvate, a natural PDK inhibitor, was added as a positive control for PDH dephosphorylation. Quantification of the western blot protein band intensities revealed that both DCA treatment and addition of pyruvate resulted in PDH dephosphorylation at serine residues 293 and 300 in *Stm* and *Mtb*-infected M1 and M2 compared to DMSO (**Figure 4A**). In summary, these data corroborate earlier findings (46, 47) that DCA treatment increases PDC activity, validating PDKs as functional targets of DCA.

Since DCA has been reported to shift metabolism from glycolysis to OXPHOS in various cell lines (32, 46, 48–50), we next evaluated whether DCA could reverse the *Stm-* and *Mtb*-induced metabolic shift towards glycolysis that was predominantly observed in human M1 macrophages (**Figure 1C**). First, we studied gene expression levels of metabolic key genes (*HK2*, *PDK2, PDK4, IDH2, SDHA*) and downstream targets of succinate accumulation (*HIF1A* and *VEGF*) (19, 22, 51), as an indication of TCA cycle activity, in human macrophages (**Figure 4B**). DCA did not significantly modify metabolic gene expression at the transcriptional level, but *VEGF* was significantly increased by DCA in *Stm*-infected M1, in accordance with increased TCA cycle activity (**Figure 4C**). Next, we studied the effect of DCA on OCR and ECAR levels in macrophages that were prestimulated with *Stm* or *Mtb* lysate (**Figure 4D**). DCA treatment resulted in increased OCR levels and decreased ECAR levels in M1, consistent with a classical DCA-induced shift from glycolysis to OXPHOS (**Figure 4E**). Strikingly, a shift from glycolysis to OXPHOS could not be detected in M2, indicating that PDC activation does not necessarily lead to glycolytic reprogramming and that this is cell-type specific.

In conclusion, DCA can be used as a chemical tool to study the effect of PDC activation on *Stm* and *Mtb* infections in human macrophages, which is accompanied by metabolic rewiring from glycolysis to OXPHOS in a M1 cell type specific manner.

### DCA Treatment Has Minor Impact on Cytokine and Chemokine Production, but Induces ROS Production by Human Macrophages

**Figure 5:**
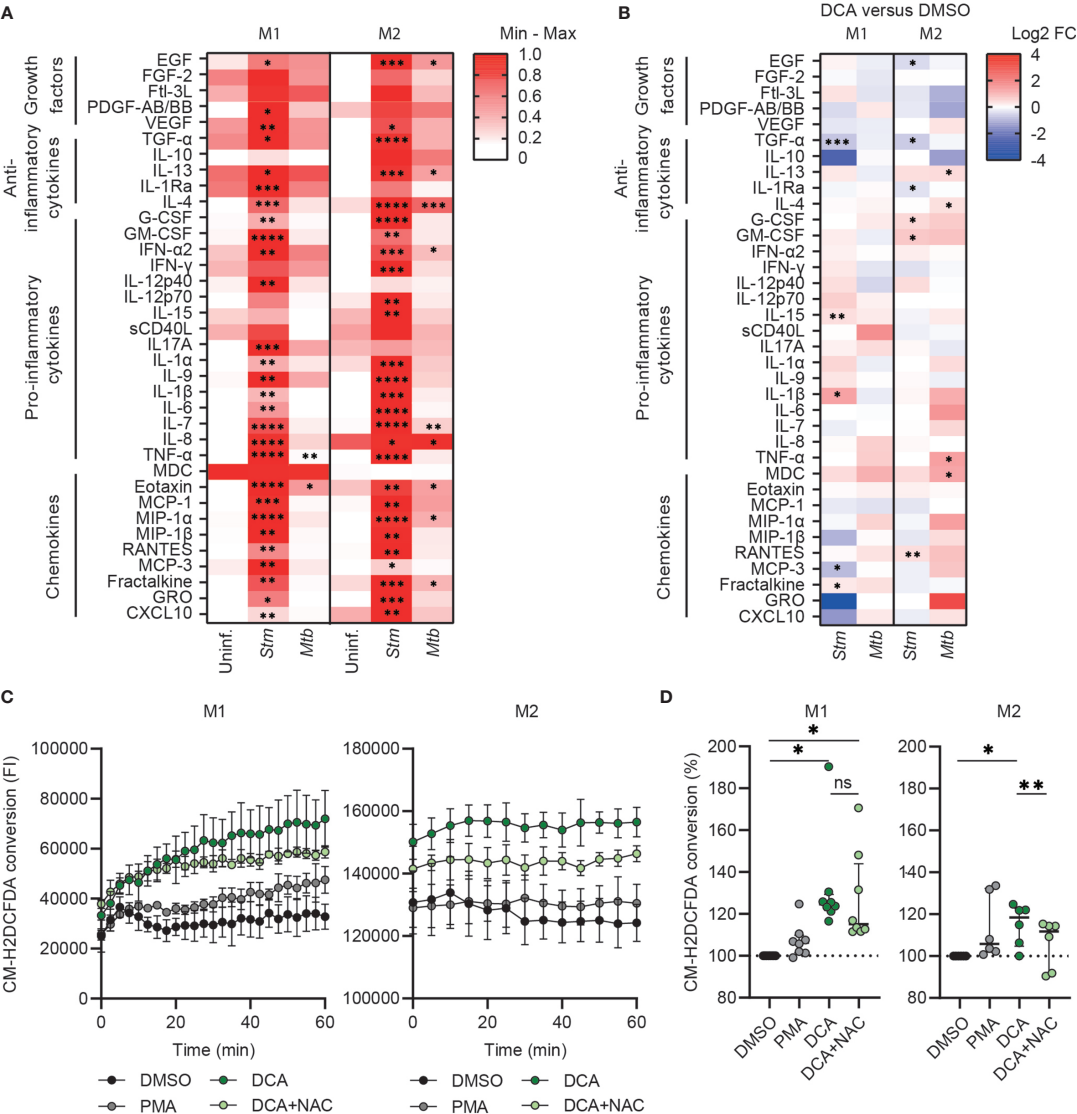
DCA induced ROS production and did not induce major cytokine/chemokine production in human macrophages. **(A)** Heatmap displaying median cytokine/chemokine levels in the supernatants of uninfected, *Stm-* or *Mtb-*infected M1 and M2 obtained from six donors. Shown are the relative secretion levels (>10 pg/ml) on a white to red color scale per cytokine/chemokine (min=0; max=1). Significant differences were tested by RM one-way ANOVA with Dunnett’s multiple comparison test against the corresponding uninfected control. **(B)** Heatmap displaying median log2 fold change (FC) cytokine/chemokine levels relative to vehicle control DMSO in the supernatants of *Stm*- or *Mtb*-infected M1 and M2 obtained from six donors. *Stm-* or *Mtb-*infected macrophages were treated with DCA (10 mM) or an equal volume of DMSO (0.1% v/v) overnight. Differences were significant by a paired t-test between DMSO and DCA. **(C, D)** ROS production in M1 (left panel) and M2 (right panel) was measured kinetically for 60 min in cells that were treated with DCA (10 mM), PMA (300 nM) or vehicle control DMSO (0.1% v/v). Data represent the fluorescent intensity (FI) at 522 nm measured kinetically of one representative experiment **(C)** and the median ± interquartile range of the area under the curves (AUC) of the FI normalized to control (i.e. DMSO) of minimally six donors **(D)**. Significant differences were tested by RM one-way ANOVA with Tukey’s multiple comparison test. *p < 0.05, **p < 0.01, ***p < 0.001, ****p < 0.001.

To study whether PDC activation can modulate effector functions of human macrophages, we assessed the production of 41 chemokines/cytokines by multiplex beads assay. Clearly, *Stm*-infection markedly induced the production of pro-inflammatory cytokines, anti-inflammatory cytokines and chemokines, which contrasted with the lower magnitude changes seen for *Mtb*-infected macrophages (**Figure 5A** and **Supplemental Table 1**). DCA treatment did not have any major impact on overall chemokine/cytokine secretion profiles of both M1 and M2, although several individual chemokines and cytokines were significantly modified by DCA in M1 and M2, dependent on the cell-type and the infection type (*Stm versus Mtb*) (**Figure 5B** and **Supplemental Table 1**).

Since PDC activation by DCA can promote OXPHOS, which is associated with mitochondrial ROS production (46, 52), we next assessed the effect of DCA on ROS production by M1 and M2. Interestingly, DCA significantly induced ROS production in both M1 and M2 and even was a more potent ROS inducer compared to positive control phorbol 12-myristate 13-acetate (PMA) (**Figures 5C, D****)**. Surprisingly, ROS production was increased immediately after administration of DCA, even before the first measurement could be taken. ROS production was still observed 1h post DCA stimulation in M1, but reached plateau levels in M2, suggesting that DCA-induced ROS production was halted. DCA-induced ROS production could be partially inhibited by N-acetylcysteine (NAC), which is a scavenger of ROS.

### DCA Treatment Induces Intracellular *Stm* Control in Human Macrophages

**Figure 6:**
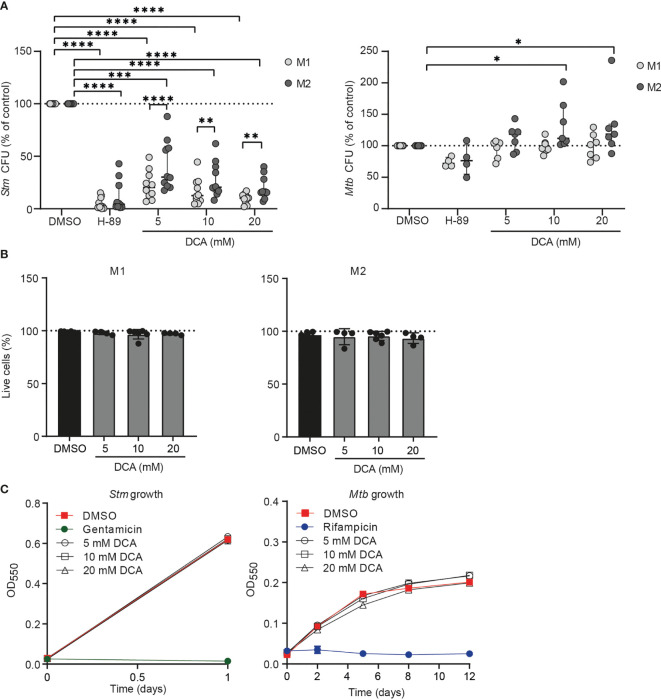
DCA markedly inhibited bacterial outgrowth of Stm but not Mtb. **(A)** *Stm*- (left panel) or *Mtb*-infected (right panel) M1 (light grey dots) or M2 (dark grey dots) were treated with H-89 (10 µM), PDK inhibitor DCA (5-20 mM) or an equal volume of vehicle control DMSO (0.1% v/v) overnight. Data represent the median ± interquartile range of ten different donors (*Stm*-infection) or seven different donors (*Mtb*-infection). CFUs are expressed as percentage of DMSO. Differences were significant by RM Two-way ANOVA with Dunnett’s-multiple comparison test to compare DMSO with DCA or with Sidak’s multiple comparison test to compare M1 with M2 in each test condition. **(B)** Percentage of live M1 (left panel) and M2 (right panel) cells (i.e. PI-negative cells) after treatment with DCA (5-20 mM) or vehicle control DMSO (0.1% v/v) overnight. Data represent the median ± interquartile range of at least four different donors. Differences were tested by RM one-way ANOVA with Dunnett’s multiple test correction against DMSO. **(C)** *Stm* (left panel) and *Mtb* growth (right panel) after addition of DCA (5-20 mM), gentamicin (50 µg/ml), rifampicin (20 µg/ml) or vehicle control DMSO (0.1% v/v) to liquid *Stm* (left panel) or *Mtb* (right panel) broth cultures. Data represent the mean optical density (OD) at 550 nm ± S.D. of triplicates of a representative experiment out of three experiments. *p < 0.05, **p < 0.01, ***p < 0.001, ****p < 0.001.

Since we showed that PDC is a target of interest for HDT and DCA activated PDC, resulting in altered metabolism and increased ROS production, we investigated whether DCA could impact bacterial control by human macrophages. Macrophages were treated with DCA or with H-89 as positive control. As expected, H-89 markedly decreased *Stm* and tended to decrease *Mtb* in M1 and M2 (41). Exposure of *Stm*- and *Mtb*-infected M1 and M2 to 5-20 mM DCA resulted in a profound decrease in *Stm* but not *Mtb* outgrowth (**Figure 6A**). Importantly, cell viability was not affected (**Figure 6B**). Interestingly, *Stm* outgrowth was significantly more inhibited by DCA treatment in M1 compared to M2, which is in agreement with the more profound effect of DCA on M1 metabolism (**Figure 4E**). Furthermore, DCA decreased the percentage of *Stm*-infected M1 and M2 cells compared to DMSO as shown by flow cytometry using DsRed-expressing *Stm* (**Supplemental Figure 4A**). Interestingly, the effect of DCA on inhibiting intracellular *Stm* outgrowth was already significant after 2 hours of DCA treatment in M2 and after 4 hours in both macrophage subsets, suggesting that DCA induces a host protective response during the early phase of infection (**Supplemental Figure 4B**).

Not unexpectedly, DCA had little effect on intracellular *Mtb* in M1 and M2 (**Figure 6A**). Even a prolonged exposure (up to 72h) of *Mtb*-infected macrophages to DCA did not result in reduction of *Mtb* CFU levels (**Supplemental Figure 4C**), suggesting that *Mtb* modulates host-signaling pathways in a manner different from *Stm*, that cannot be redressed by DCA. Potential direct antibiotic properties of DCA were excluded by adding DCA to *Stm* and *Mtb* cultures in the absence of human cells, showing that DCA acts *via* host-directed pathways in human macrophages to control intracellular *Stm* (**Figure 6C**).

Taken together, our data suggest that DCA inhibits intracellular *Stm* infection, but not *Mtb* infection, in a host-directed manner. DCA reduced *Stm* levels rapidly after administration, suggesting that DCA is a potent molecule for treatment against *Stm*.

## Discussion

Physiological metabolic modulation of phagocytes is essential to effective host control of intracellular bacterial pathogens (15). Here, we show that human M1 and M2 adapt their metabolic profile upon *Stm* and *Mtb* lysate stimulation and *Stm* infection by increasing glycolysis. We identified metabolic key genes, PDKs, as novel targets for HDT against *Stm* and report DCA as a functional chemical tool to study PDK inhibition in the context of bacterial infections. Furthermore, we report cell-specific effects of DCA on host cell metabolism and chemokine and cytokine production and we show major and rapid effects of DCA on *Stm* outgrowth in infected human macrophages. DCA has been described to induce bacterial clearance in the peritoneum of septic mice (53), and to our knowledge, this is the first report of a host-directed anti-bacterial effect of DCA on *Stm*.

Rapid upregulation of glycolysis is a hallmark of intracellular bacterial infections and is thought to rapidly provide energy to sustain biosynthesis of inflammatory molecules during the initial infection stage (9–16). However, most studies have been performed in mouse models, and the number of studies that assessed induction of glycolysis by bacterial infections in human cellular models is limited. Only few studies have reported that human macrophages do not increase glycolysis in response to LPS and intracellular trypanosomatid parasites (*Leishmania donovani*, *L*. *amazonensis* and *T*. *cruzi*) (35, 36). In the current study, M1 and M2 subsets increased their glycolytic metabolism upon infection with *Stm*, as demonstrated by increased extracellular acidification rates and lactate production and reduced succinate dehydrogenase complex subunit A (SDHA) mRNA levels. Importantly, and in agreement with findings from others (35, 36), our work does not confirm the reported metabolic divergence between M1 and M2 in mice, where glycolysis is the dominant metabolic pathway in M1 and OXPHOS is the dominant metabolic pathway in M2 in meeting general energy demands (27–29, 54). In fact, in support of our data, LPS stimulation enhanced the glycolytic pathway already after 3 hours in human M1 and M2 macrophages that were differentiated from monocytes by using M-CSF and IFNγ or M-CSF, respectively (55). Moreover, glycolysis was induced in human alveolar macrophages upon *Mtb* infection and in human monocytes upon BCG infection (10, 56). Here, we report that stimulation with *Mtb* lysate increased the ECAR/OCR ratio to similar levels as *Stm* lysate. In contrast to infection with live *Stm*, live *Mtb* infection was not able to increase lactate levels in M1, suggestive of decreased glycolytic function in *Mtb*-infected cells compared to *Stm*-infected cells. This supports the findings by others that live *Mtb* may actively suppress the glycolytic pathway in M1 (37, 38).

Inhibition of glycolysis by 2-DG inhibited *Stm* but not *Mtb* outgrowth in our macrophage model. Importantly, our data in primary human macrophages are in contrast with published data from mouse BMDMs in which inhibition of glycolytic flux by 2-DG or galactose reduced IFN-γ-dependent control of intracellular *Mtb* (39). Moreover, mice treated *in vivo* with 2-DG had higher *Mtb* bacterial burdens in their lungs after 14 days (40). Although 2-DG is classically reported to inhibit glycolysis, it also inhibits OXPHOS when used in high concentrations that are similar to the ones in our study (up to 10 mM) (44). Furthermore, the inhibitory effect of 2-DG on *Stm* and *Mtb* infection could be dependent on other pathways than its impairment of the glycolytic pathway or OXPHOS, as has already been suggested for *Legionella pneumophila* (43). In agreement with others, we thus propose caution in the interpretation of data generated using 2-DG in the context of glycolysis (44, 57, 58). Alternatively, substitution of glucose for galactose can provide a more reliable method to inhibit glycolysis (44). In our work substitution of D-glucose for D-galactose tended to increase intracellular *Mtb*, in agreement with reported data in mouse BMDM (39), and in line with the hypothesis that glycolysis is required for protective host responses against *Mtb*. Importantly, substitution of D-glucose for D-galactose significantly decreased intracellular levels of *Stm*, suggesting that the classical “host-protective” role of glycolysis during bacterial infections does not apply to *Stm* in human macrophages.

PDKs are considered gate-keeping kinases between glycolysis and OXPHOS and chemical inhibition of PDKs by DCA resulted in a significant reduction of intracellular *Stm* outgrowth in human macrophages. DCA is a potent small molecule in clinical use for the treatment of lactic acidosis and metabolic disorders (59) and has gained serious interest as anti-cancer therapeutic in the past decade (60). DCA has passed phase I/II toxicity testing in humans, which can accelerate the translation towards to clinic application for other diseases. Administration of DCA displayed a broad spectrum regarding toxicity and safety, ranging from well-tolerance (61) and common gastrointestinal side-effects (62), to severe side-effects like peripheral neuropathy (63, 64). Despite reported toxicities, DCA has not lost interest as potential therapeutic, and severe side-effects may be suppressed with adjuvant therapy (65). Tolerance of DCA in patients infected with Salmonella remains to be determined and is crucial for its applicability as HDT compound for the treatment of Salmonella infections, which has a mild disease course in most patients, but can be persistent and even lethal in others (66, 67). Building upon this potential, we have identified PDKs as potential novel host targets for HDT to treat *Stm* infections. Of note in this context, several novel, potentially safer, DCA derivatives have been synthesized recently (59).

DCA classically induces a metabolic shift from glycolysis to OXPHOS, which was reported in cancer cell lines (32, 46, 48–50) and mouse embryonic stem cells (68). Similarly, DCA significantly decreased ECAR levels in human macrophages differentiated with M-CSF or M-CSF with IFN-y (55). Here, we report a similar result using DCA in M1 stimulated with *Stm* or *Mtb* lysate, but not in M2. Differences in ECAR levels between M-CSF-derived macrophages published by Chiba et al. and our M-CSF-derived M2 may be explained by differences in DCA concentrations tested (20 mM *versus* 10 mM in our study), or by the use of pre-stimulation with *Stm* or *Mtb* lysate in our study. In addition to decreased ECAR levels, we observed increased OCR levels immediately after DCA treatment in M1, indicative of a metabolic shift from glycolysis to OXPHOS. Interestingly, we observed increased levels of *VEGF* mRNA levels in *Stm*-infected M1 and not in M2. Since succinate has been associated with HIF-1α stabilization, we hypothesize that the increased *VEGF* mRNA levels are a consequence of increased flux into the TCA cycle leading to succinate accumulation. Thus, importantly, we show that DCA induces cell-type specific responses, but not general metabolic rewiring in all cell types. Since DCA-induced metabolic effects were only observed in M1 and not in M2, we propose that improved control of *Stm* was not caused by a metabolic shift from glycolysis to OXPHOS. While not investigated here, DCA treatment may have increased or decreased accumulation of specific TCA cycle metabolites that modulate innate immune responses of macrophages to *Stm*, such as succinate (69), or may have deprived the cells from host carbon sources that can be exploited by *Stm*, for instance lactate (70).

In previous studies, DCA induced the functional polarization of murine macrophages towards an anti-inflammatory phenotype based on cytokine production profiles (71, 72). Similarly, in human cells, DCA decreased IL-1β and IL-10 levels in LPS-stimulated human THP-1 cells (73) and IL-6 levels in LPS-stimulated human macrophages (55). In contrast with these studies, we did not observe significant DCA-inhibiting effects on IL-1β, IL-10 and IL-6 secretion by *Stm*- or *Mtb*-infected human macrophages, possibly because infection alone already strongly skewed the cytokine response (**Figure 5A**). Generally, we observed that the effect of DCA on chemokine/cytokine secretion was dependent on both cell-type (M1 *versus* M2) and infection (*Stm versus Mtb*). We therefore conclude that, based on their cytokine secretion profile, DCA was not able to (re)polarize *Stm*- or *Mtb*-infected human M1 and M2 towards an anti-inflammatory phenotype.

Potential mechanism by which DCA induces *Stm* control in human macrophages is through increasing ROS production, as DCA clearly induced ROS in both M1 and M2. In agreement with our data, DCA increased intracellular ROS production by tumor cells, head and neck cancer cells and multiple myeloma cells (46, 52, 74). Although ROS production appears a host strategy to cope with *Stm* (75–77), it can also promote *Stm* survival, and thus ROS acts as a double-edged sword in the perspective of the host and *Stm* (78). While DCA-induced ROS production has been suggested to originate from increased OXPHOS activity in the mitochondria (46, 74), we cannot not exclude other sources of ROS production based on our data. Increased ROS production in M2, despite no detectable changes in OXPHOS in these cells, in fact might suggest that ROS may have originated from other sources, for instance NADPH oxidase-dependent ROS production of phagosomal origin (79). Given that the type III secretory system of Salmonella interferes with host NADPH oxidase trafficking to the phagosome [as reviewed in (80)] to evade ROS-induced killing by phagocytes, we speculate that NADPH localization may be restored by DCA treatment. This effect of DCA on NADPH oxidase localization and the effect of ROS production on intracellular *Stm* control requires future investigation.

In the current study, we showed that PDK expression is modulated during *Stm* infections and can be targeted by DCA to improve host control against *Stm* infections. Furthermore, we evaluated effects of DCA on *Stm* from a host perspective. Whether the effect of DCA on *Stm* control is caused by limited pyruvate access as carbon source remains to be determined, but may explain why DCA did not decrease intracellular *Mtb* levels. Together, our data highlight differences in survival of two pathogenic bacteria that can use the same host niche, and show the clinical potential of DCA as novel HDT treatment against *Stm*.

## Materials And Methods

### Reagents and Antibodies

Dimethyl sulfoxide (DMSO), 2-deoxy-D-glucose (2-DG), D-(+)-glucose, D-(+)-galactose, sodium dichloroacetate (DCA), sodium pyruvate, sodium L-lactate, H-89 dihydrochloride hydrate (H-89), and phorbol 12-myristate 13-acetate (PMA) were obtained from Sigma-Aldrich (Zwijndrecht, The Netherlands). Carbonyl cyanide 4-(trifluoromethoxy)phenylhydrazone (FCCP) was obtained from Seahorse Bioscience (Billerica, MA, USA). FITC Anti-human CD14 monoclonal (M5-E2) and A647 anti-human CD163 monoclonal (RM3/1) were purchased from Sony Biotechnology Inc. (San Jose, California, USA). PE anti-human CD11b monoclonal (ICRF44) was obtained from BD Bioscience (Vianen, The Netherlands). Anti-human PDH-E1a (pSer^293^) and anti-human PDH-E1a (pSer^300^) polyclonal antibodies were purchased from Merck Millipore (Amsterdam, The Netherlands). Anti-human vinculin monoclonal (hVIN-1) was purchased from Sigma-Aldrich and anti-human PDH monoclonal (9H9AF5) from Thermo Fisher Scientific (Landsmeer, The Netherlands). Secondary stabilized peroxidase conjugated antibodies goat anti-rabbit IgG (H+L) and goat anti-mouse IgG (H+L) were purchased from Thermo Fisher Scientific.

### Cell Culture

Primary human macrophages were obtained as described earlier (25). Briefly, human peripheral blood mononuclear cells (PBMCs) were isolated from buffy coats obtained from healthy donors with informed consent by density gradient centrifugation over Ficoll/amidotrizoaat (Pharmacy, LUMC, The Netherlands). CD14+ monocytes were isolated by magnetic cell sorting using anti-CD14-coated microbeads (Miltenyi Biotec, Auburn, CA) and cultured at 37°C/5% CO_2_ in Gibco Roswell Park Memorial Institute (RPMI) 1640 medium (Thermo Fisher Scientific) supplemented with 10% FBS, 2 mM L-Alanyl-L-Glutamine (PAA, Linz, Austria), 100 units/ml penicillin, 100 µg/ml streptomycin and either 5 ng/ml granulocyte-macrophage colony-stimulating factor (GM-CSF, Thermo Fisher Scientific) to promote M1-differentiation or 50 ng/ml macrophage colony-stimulating factor (M-CSF, R&D Systems, Abingdon, United Kingdom) to promote M2-differentiation. After 6 days of differentiation, macrophages were detached by trypsinization and gentle scraping, counted and seeded for downstream application in RPMI 1640 medium (10% FBS and 2 mM L-Alanyl-L-Glutamine). For experiments, cells were incubated overnight at 37°C/5% CO_2_. In parallel, M1 and M2 macrophage phenotypes were validated based on surface marker expression (M1: CD14^low^, CD163^low^, CD11b^high^; M2: CD14^high^, CD163^high^, CD11b^low^) by flow cytometry based on cytokine production (IL-10 and IL-12) following overnight LPS stimulation (100 ng/ml) as described before (81). Cells were plated on round-bottom 96 wells plates in a concentration of 75,000 cells/well in RPMI 1640 and washed in 150 µl PBS/0.1% BSA. Fc receptors were blocked by adding 50 µl of 5% human serum (Sanquin, Amsterdam, the Netherlands) for 10 minutes. Cells were washed and stained with antibodies against CD14, CD163 and CD11b for 30 minutes at 4°C in the dark, washed twice and fluorescence intensity was measured on a Accuri™ C6 flow cytometer (BD Bioscience).

### Bacterial Culture

DsRed-expressing-*Salmonella enterica* serovar Typhimurium (*Stm*) strain SL1344 (42) was recovered from frozen stock and cultured at 37°C in Difco™ Luria-Bertani (LB) broth (BD Bioscience) containing 100 µg/ml ampicillin (Sigma-Aldrich). DsRed-expressing-*Mycobacterium tuberculosis* H37Rv (42) was cultured at 37°C in Difco™ Middlebrook 7H9 broth (BD Bioscience, Vianen, The Netherlands) containing 10% ADC (Becton Dickinson, Breda, The Netherlands), 0.5% Tween-80 (Sigma-Aldrich) and 2% Glycerol (Sigma-Aldrich). *Stm* and *Mtb* lysates were generated by harvesting log-phase liquid *Stm* and *Mtb* cultures. Cultures were washed twice and resuspended in PBS. 1 ml glass beads (0.1 mm, BioSpec Products, Breda, The Netherlands) were added to 1 ml bacterial suspension in PBS and lysates were generated using the Mini-BeadBeater-1 (BioSpec Products). After 5 minutes of incubation, cell lysates were transferred to a 1.5 ml Eppendorf tubes and protein concentration was quantified by Pierce™ BCA protein assay kit according to manufacturer’s instructions (Thermo Fisher Scientific).

### Seahorse Real-Time Extracellular Flux Analysis

Oxygen consumption rate (OCR) and extracellular acidification rate (ECAR) were determined as a measure of mitochondrial respiration and glycolysis, respectively, by using the Agilent Seahorse XFe96 Extracellular Flux Analyzer (Seahorse Bioscience) according to manufacturer’s instructions and as described before (82). Briefly, cells were plated in a Seahorse XF cell culture microplate at a density of 30,000 cells/well and incubated overnight at 37°C/5% CO_2_ in RPMI 1640 medium (10% FBS, 2 mM L-Alanyl-L-Glutamine). Cells were stimulated with 1 or 10 µg/ml *Stm* or *Mtb* lysate and incubated for 4 hours at 37°C/5% CO_2_. Plates were washed with glucose-free RPMI 1640 medium (Sigma-Aldrich) containing 5% FBS and incubated for 1h at 37°C without CO_2_. To determine the effect of chemical compounds on OCR and ECAR, cells were stimulated with 10 µg/ml *Stm* or *Mtb* lysate and inserted in the Seahorse XFe96 Analyzer directly. After insertion into the Seahorse XFe96 Analyzer, wells were sequentially injected with final concentrations of 10 mM D-glucose with or without chemical compounds, 1 µM oligo (ATP synthase inhibitor) and 2 µM FCCP (mitochondrial uncoupling agent) after 18, 54 and 72 minutes, respectively. Basal glycolysis was calculated as the difference between lowest ECAR measurement pre-D-glucose injection and highest ECAR measurement post-D-glucose injection. The glycolytic capacity was calculated as the difference between lowest ECAR measurement pre-D-glucose injection and highest ECAR measurement post-oligo injection. OXPHOS was calculated as the difference between highest OCR measurement pre-oligo injection and lowest OCR measurement post-oligo injection. Maximal respiration was calculated as the difference between lowest OCR measurement pre-FCCP injection and highest OCR measurement post-FCCP injection. Basal glycolysis was divided by OXPHOS to calculate ECAR/OCR ratios.

### Macrophage Infection

Ongoing *Stm* cultures were diluted one day before infection and again at 2-3 hours prior to infection to reach log phase (OD_600_ between 0.4-0.6) during infection. *Mtb* cultures were diluted one day prior to infection to reach log phase (OD_600_ between 0.4-0.6) during infection. Cells were infected with *Stm* or *Mtb* with a multiplicity of infection (MOI) of 10. Accuracy of the MOI was validated by plating serial dilutions of the *Stm* inoculum on Difco™ LB agar plates (BD Bioscience) or the *Mtb* inoculum on Difco™ Middlebrook 7H10 agar (BD Bioscience) plates containing 10% OADC (Becton Dickinson) and 5% glycerol. Cells seeded 1 day prior to infection in flat-bottom 96-well plates were inoculated with 100 µl bacterial suspension and cells seeded in flat-bottom 24-well plates with 500 µl bacterial suspension in RPMI 1640 (10% FBS, 2 mM L-Alanyl-L-Glutamine). Plates were centrifuged for 3 minutes at 800 rpm and incubated for 20 minutes for *Stm* infections and 1 hour for *Mtb* infections at 37°C/5% CO_2_. Extracellular bacteria were removed by washing and incubation with fresh RPMI 1640 (10% FBS, 2 mM L-Alanyl-L-Glutamine) containing 30 µg/ml gentamicin sulphate (Lonza BioWhittaker, Basel, Switzerland) for 10 minutes and cells were incubated in gentamicin sulphate-containing medium (5 µg/ml) in the presence or absence of chemical compounds or an equal amount of vehicle control DMSO (0.1% v/v) for 4 hours or overnight at 37°C/5% CO_2_.

### Lactate Assay

Cell supernatants were harvested and kept at -20°C until further use. 10 µl of 100 mM sodium l-lactate standard or sample was added in triplicate to a flat bottom 96-well plate (Greiner Bio-One, Alphen a/d Rijn, The Netherlands). 200 µl of reaction mix (0.74 mM NAD, Roche Applied Science; 0.4 mM glycine, Sigma-Aldrich; 0.4 M hydrazine hydrate, Sigma-Aldrich) and 2 µl of three times diluted L-Lactate Dehydrogenase (LDH) from rabbit muscle (Roche Applied Science, Bayern, Germany) was added to each well to allow conversion of lactate to pyruvate by LDH and trapping of the newly formed pyruvate: Lactate + NAD^+^ <–> Pyruvate + NADH + H^+^. Plates were incubated on a shaker for 90 minutes at RT and NADH levels were measured spectrophotometrically at 340 nm using a SpectraMax i3x plate reader (Molecular Devices, CA, USA) before and after incubation as an indicator of lactate production.

### CFU Assay

Intracellular bacterial burden was determined by colony forming unit (CFU) assays as described previously (81). Cells were washed once with PBS and lysed in 0.05% sodium dodecyl sulfate (SDS) solution (Thermo Fisher Scientific). Serially diluted cell lysates were plated on LB agar (*Stm*) or on 7H10 agar containing 10% OADC and 5% Glycerol (*Mtb*). Plates were incubated at 37°C and CFUs were counted from triplicate wells.

### Cellular Toxicity Assay

The number and percentage of dead cells based on plasma membrane integrity of the adherent cell population was quantified by analysis of microscopy images. Cells in 96-well flat bottom plates (30,000 cells/well) were stained with 2 µg/ml propidium iodide (PI, Sigma-Aldrich) and 2 µg/ml Hoechst 33342 (H3570, Sigma-Aldrich) in 40 µl/well phenol red-free RPMI 1640 (Sigma-Aldrich) supplemented with 10% FBS and 2 mM L-Alanyl-L-Glutamine and incubated for 5 minutes at room temperature (RT). Cells were imaged using a Leica AF6000 LC fluorescence microscope (Leica Microsystems, Wetzlar, Germany) combined with a 10x dry objective. Total and dead cell numbers were quantified by respectively counting the nuclei and the number of PI-positive cells using Fiji/ImageJ software (83).

### Cell-Free Bacterial Growth Assay

Compounds were diluted in Difco™ LB broth or in Difco™ Middlebrook 7H9 broth and added (100 µl/well) to 100 µl/well bacterial culture (OD_600_ of 0.1) in flat bottom 96-well plates. Gentamycin (50 µg/ml) and rifampicin (20 µg/ml) were added as positive controls to inhibit *Stm* and *Mtb* growth, respectively. Absorbance was measured directly after plating and after 16-18 hours for *Stm* growth and at day 2, 5, 8 and 12 for *Mtb* growth at 550 nm on a Mithras LB 940 plate reader (Berthold Technologies, Bad Wildbad, Germany).

### Fluidigm Real-Time Quantitative Polymerase Chain Reaction (qPCR)

300,000 cells/well in a flat bottom 24 wells plate were lysed in 350 µl TRIzol™ reagent (Thermo Fisher Scientific). RNA was isolated according to manufacturers’ instructions and diluted to 50 ng/µl. cDNA and 14-cycle preamplification was performed according to Fluidigm protocols (Biomark Fluidigm, South San Francisco, CA, United States). Briefly, cDNA was synthesized by addition of 1 µl reverse transcription master mix to 3 µl of RNase-free water and 1 µl of 50 ng/µl RNA per sample, and incubated using the following thermal protocol: 5 min at 25°C, 30 min at 42°C, 5 min at 85°C and then stored at -20°C until use. For preamplication, 1.25 µl of cDNA was added to 1 µl of Preamp Fluidigm Master Mix, 1.25 µl of Pooled TaqMan primer mix and 1.5 µl of RNase-free water per sample, and incubated using the following thermal protocol: 2 min at 85°C, 14 cycles of 15s at 95°C and 4 min at 60°C and stored at -20°C until use. For quantitative real-time PCR reactions, 1.35 µl preamplified cDNA, 1.5 µL 2X Taqman Gene Expression PCR Master Mix and 0.15 µL 20X GE Sample Reagent was used per reaction. Flex Six fluidics chips were primed with control line fluid and loaded with 3 µl preamplified samples in the appropriate inlets. All Real-Time PCR reactions were performed in the BioMark real-time PCR system using the GE FLEXSix Standard v1 thermal protocol. Cycle threshold (Ct) values were calculated using BioMark Real-time PCR Analysis software. Changes of expression values were calculated as the log2 Fold Change (FC) between the target gene and the reference gene *GAPDH*. The following FAM-MGB Taqman primer sets were used: *GAPDH* (Hs02758991_g1), *PDK1* (Hs01561847_m1), *PDK2* (Hs00176865_m1), *PDK3* (Hs00178440_m1), *PDK4* (Hs01037712_m1), *PDP1* (Hs01081518_s1), *PDP2* (Hs01934174_s1), *HK2* (Hs00606086_m1), *SDHA* (Hs00188166_m1), *IDH2* (Hs00953879_m1), *HIF1A* (Hs00153153_m1) and *VEGFA* (Hs00900055_m1).

### Western Blot Analysis

Cells (300,000 cells/well in 24-wells plates) were lysed using 100 µl/well EBSB buffer (10% v/v glycerol, 3% SDS, 100 mM Tris-HCl, pH 6.8) supplemented with cOmplete™ EDTA-free protease inhibitor cocktail (Sigma-Aldrich) and phosphatase inhibitor (PhosSTOP EASYpack Sigma-Aldrich) in the concentration of one tablet each per 10 ml. Samples were boiled for 10 minutes at 95°C and stored at -20°C until use. Sample concentrations were determined using Pierce™ BCA protein assay kit (Thermo Fisher Scientific) according to manufacturer instructions and diluted to equal concentrations in Laemmli sample buffer (Biorad) containing 10% β-mercaptoethanol (Sigma-Aldrich). 12.5 µl of sample per lane was loaded on a 15-well 4–20% Mini-PROTEAN^®^ TGX™ Precast Protein Gel (Bio-Rad Laboratories, Veenendaal, the Netherlands) and Amersham ECL Rainbow Molecular Weight Marker (Sigma-Aldrich) was added as reference. Proteins were transferred to methanol-activated Immun-Blot PVDF membranes (Biorad) in Tris-glycine buffer (25 mM Tris, 192 mM glycine and 20% methanol). Membranes were blocked for 1 hour in polysorbate 20 tris-buffered saline (TTBS) supplemented with bovine serum albumin fraction V (BSA, 5% w/v, Roche Diagnostics, Almere, The Netherlands) and incubated with the indicated antibodies in 5% BSA/TTBS overnight at 4°C. Membranes were washed for 45 minutes in TTBS and stained with secondary antibodies in 5% BSA/TTBS for 2 hours at RT. Membranes were washed for 30 minutes with TTBS prior to revelation using enhanced chemiluminescence (ECL)™ Prime Western Blotting System reagent (GE Healthcare, Hoevelaken, The Netherlands). Imaging was performed on a ChemiDoc XRS+ (Bio-Rad). Protein bands were quantified using ImageJ/Fiji software (83) and normalized to vinculin.

### Reactive Oxygen Species (ROS) Assay

Procedure was performed as described previously (81). Cells (40,000 cells/well) were seeded in black ultra-thin clear flat bottom 96-well plates (Corning) in RPMI 1640 (10% FBS, 2 mM L-Alanyl-L-Glutamine) and incubated overnight at 37°C/5% CO_2_. Cells were washed with 150 µl PBS/well and RPMI 1640 without phenol red was added and background fluorescence was measured using a SpectraMax i3x plate reader (λ ex 493 nm and λ em 522 nm) at 37°C. Cells were washed with 150 µl PBS/well. 50 µl/well of 5 µM CM-H2DCFDA, a general ROS probe, in RPMI 1640 without phenol red was added for 30 minutes at 37°C/5% CO_2_. Cells were washed with 100 µl PBS/well and 100 µl/well RPMI 1640 without phenol red was added containing DMSO (0.1% v/v), DCA (10 mM) or PMA (300 nM) as positive control. ROS production was measured kinetically for 60 minutes at 37°C by measuring fluorescence at 522 nm using the SpectraMax i3x.

### Cytokine and Chemokine Secretion by Enzyme-Linked Immuno Sorbent Assay (ELISA) and Multiplex Beads Assay

Cell supernatants were collected and sterilized by centrifugation using a 96-well filter plate containing a 0.2 µm PVDF membrane (Corning). IL-12p40/p70 production was quantified using a human IL-12p40/p70 Enzyme-Linked Immuno Sorbent Assay (ELISA) kit (Thermo Fisher Scientific) according to manufacturer’s instructions. Secretion levels of forty-one cytokine/chemokine analytes were quantified using the MilliPlex Human Cytokine/Chemokine magnetic bead premixed 41-plex kit (Millipore Billerica, MA, USA) according to manufacturer’s instructions. The following analytes were measured on a Bio-Plex 100 with Bio-Plex ManagerTM software v6.1 (Biorad): sCD40L, EGF, FGF-2, Flt3 ligand, Fractalkine (CX3CL1), G-CSF, GM-CSF, GRO (CXCL1), IFN-γ, IFN-α2, IL-1α, IL-1β, IL-1RA, IL-2, IL-3, IL-4, IL-5, IL-6, IL-7, IL-8 (CXCL8), IL-9, IL-10, IL-12p40, IL-12p70, IL-13, IL-15, IL-17a, IP-10 (CXCL10), MCP-1 (CCL2), MCP-3 (CCL7), MDC (CCL22), MIP-1α (CCL3), MIP-1β (CCL4), PDGF-AB/BB, RANTES (CCL5), TGF-α, TNF-α, TNF-β, VEGF, Eotaxin (CCL11) and PDGF-AA. Cytokines that were produced in quantities lower than 10 pg/ml in all treatment conditions were excluded from analysis.

### Statistical Analysis

Data were tested with a paired sample t-test when comparing two groups and RM one-way ANOVA followed by Dunnett’s multiple comparison test, unless mentioned otherwise, when comparing three or more groups. Statistical tests were considered significant when p < 0.05 at 95% confidence interval. All statistical analyses were carried out using GraphPad Prism 8 software (Graphpad Software, San Diego, CA, USA).

## Data Availability Statement

The raw data supporting the conclusions of this article will be made available by the authors, without undue reservation.

## Ethics Statement

The studies involving human participants were reviewed and approved by Institutional Review Board of the Leiden University Medical Center, The Netherlands. The patients/participants provided their written informed consent to participate in this study.

## Author Contributions

TO and CD devised the project and the main conceptual ideas. MH and FV contributed to the design and implementation of the research. CD, GS, JE, SV, and MH performed the experiments and analyzed the experimental data. KW contributed to preparation of materials. CD and TO wrote the manuscript. TO supervised the project. All authors contributed to the article and approved the submitted version.

## Funding

The study received support from the Netherlands Organization for Scientific Research (NWO-TOP Grant Agreement No. 91214038); and the NWO-TTW NACTAR grant (Grant Agreement No. 16444). The funders had no role in study design, data collection and analysis, decision to publish, or preparation of the manuscript.

## Conflict of Interest

The authors declare that the research was conducted in the absence of any commercial or financial relationships that could be construed as a potential conflict of interest.

## Publisher’s Note

All claims expressed in this article are solely those of the authors and do not necessarily represent those of their affiliated organizations, or those of the publisher, the editors and the reviewers. Any product that may be evaluated in this article, or claim that may be made by its manufacturer, is not guaranteed or endorsed by the publisher.
