# Supplementary material for: Pyruvate Dehydrogenase Kinase Inhibitor Dichloroacetate Improves Host Control of Salmonella enterica Serovar Typhimurium Infection in Human Macrophages
Source: Front Immunol. 2021 Sep 6;12:739938. doi: 10.3389/fimmu.2021.739938 (PMC8450447; doi:10.3389/fimmu.2021.739938)
Supplement: Supplementary file 1 [file DataSheet_1.pdf]

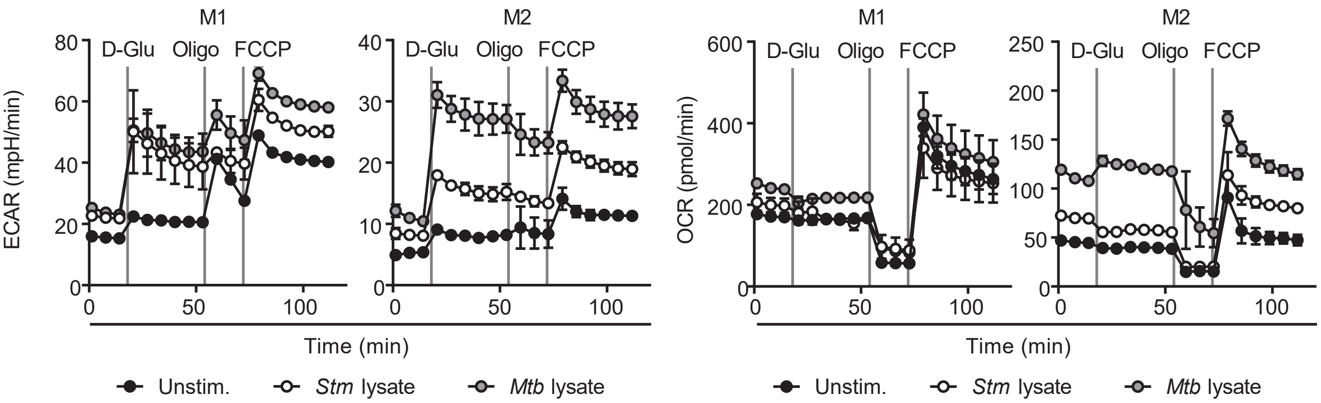

Supplemental Figure 1. *Stm* and *Mtb* induced a more glycolytic phenotype in human macrophages compared to uninfected cells. M1 and M2 were unstimulated (unstim.) or stimulated with 10 µg/ml *Stm* or *Mtb* lysate for 4 hours and inserted in the Seahorse analyzer. D-Glucose (10 mM), oligomycin (oligo, 1 µM) and carbonyl cyanide 4-(trifluoromethoxy)phenylhydrazone (FCCP, 2 µM) were sequentially injected after 18, 54 and 72 minutes, respectively. ECAR and OCR profiles of one representative donor out of four donors analyzed. Data represent the mean ± S.D. of triplicates.
