# Supplementary material for: Pyruvate Dehydrogenase Kinase Inhibitor Dichloroacetate Improves Host Control of Salmonella enterica Serovar Typhimurium Infection in Human Macrophages
Source: Front Immunol. 2021 Sep 6;12:739938. doi: 10.3389/fimmu.2021.739938 (PMC8450447; doi:10.3389/fimmu.2021.739938)
Supplement: Supplementary file 3 [file DataSheet_3.pdf]

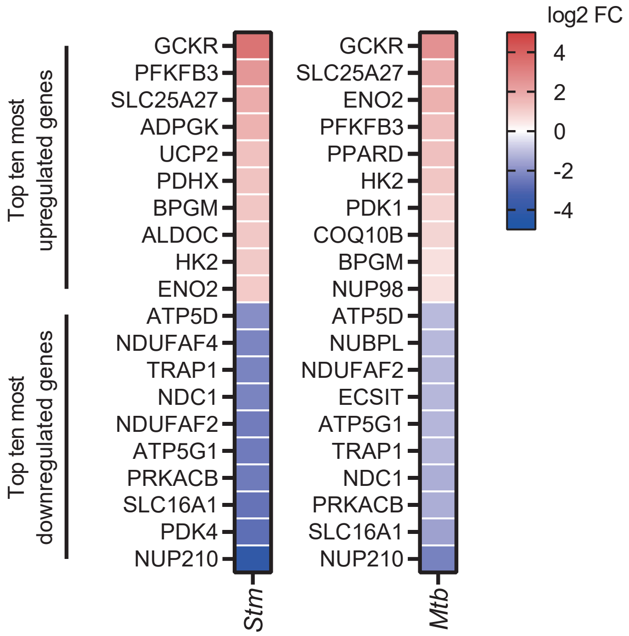

Supplemental Figure 3. Top ten most upregulated and top ten most downregulated metabolic genes in human macrophages upon *Stm* and *Mtb* infection. Heatmap displaying median log2 fold changes (FC) of the top ten most upregulated or downregulated genes involved in 'glucose metabolism' and 'the citric acid (TCA) cycle and respiratory electron transport' in M2 obtained from six donors 18 hours post *Stm* or *Mtb* infection (MOI 5) compared to uninfected controls. Genes that were significantly up- or downregulated by limma-voom (adjusted p-value < 0.05) are shown using a red to white to blue color scale. Data obtained from Blischak *et al.* (45).
