# Supplementary material for: Pyruvate Dehydrogenase Kinase Inhibitor Dichloroacetate Improves Host Control of Salmonella enterica Serovar Typhimurium Infection in Human Macrophages
Source: Front Immunol. 2021 Sep 6;12:739938. doi: 10.3389/fimmu.2021.739938 (PMC8450447; doi:10.3389/fimmu.2021.739938)
Supplement: Supplementary file 4 [file DataSheet_4.pdf]

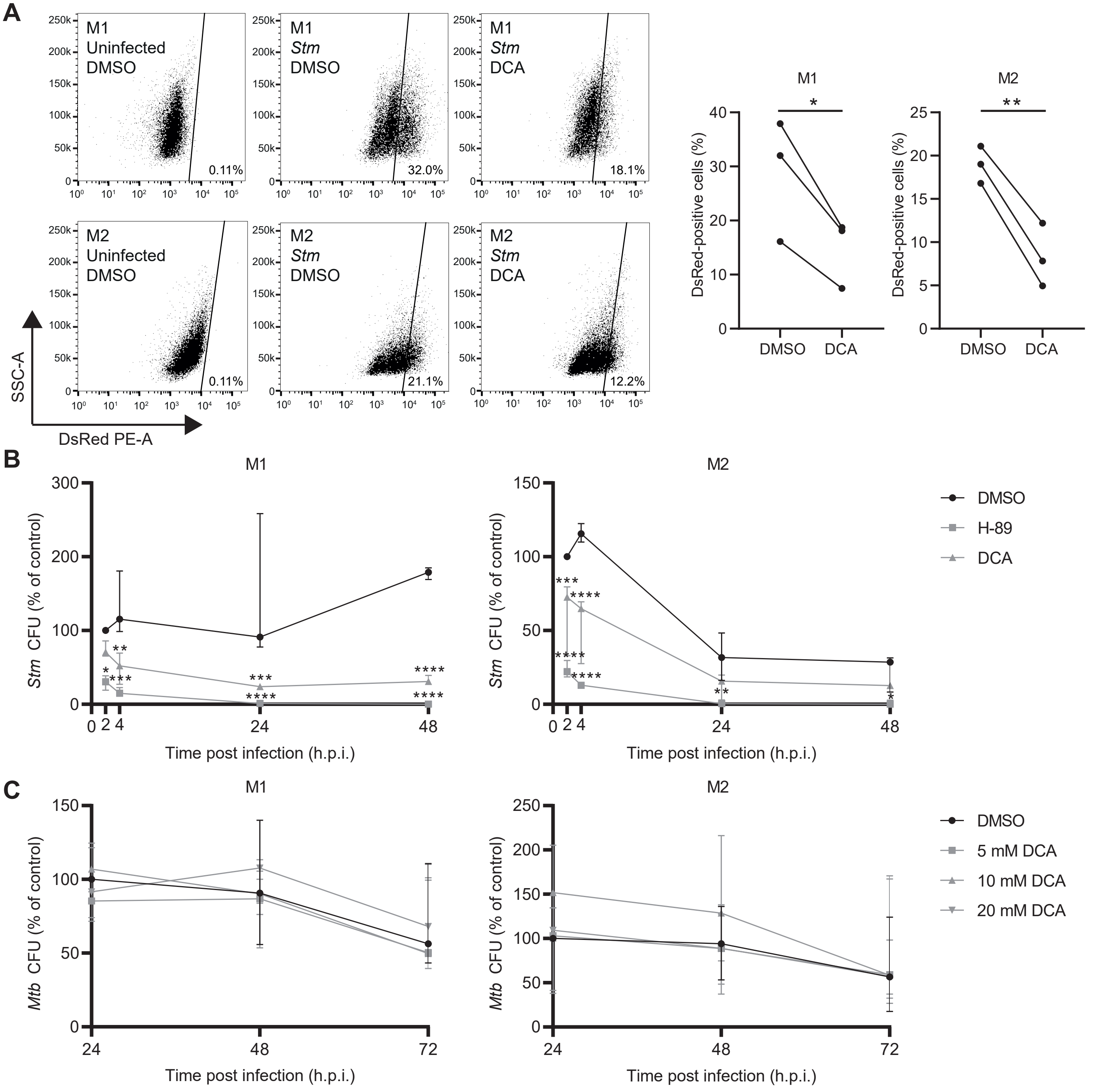


**Supplemental Figure 4. DCA markedly inhibited bacterial outgrowth of *Stm* but not *Mtb*.** (**A**) Flow cytometer plots of *Stm*-infected M1 and M2 that were treated with 10 mM DCA or vehicle control DMSO (0.1% v/v) overnight. Shown are the side scatter area on the y-axis and the DsRed-PE area on the x-axis of one representative donor (left panel) and the quantified bacterial load (DsRed-positive cells) as percentage of the total cell number (right panel). Lines connect data points from the same donor, with a total of three donors tested. Cells were gated for DsRed-positivity based on uninfected control cells. Differences were significant by a paired t-test. (**B**) *Stm* CFUs following treatment with H-89 (10 µM), DCA (10 mM) or vehicle control DMSO (0.1% v/v) at 2, 4, 24 or 48 h.p.i. Data represent the median ± range of three donors. CFUs are expressed as percentage of DMSO. Differences were significant by RM Two-way ANOVA with Dunnett's-multiple comparison test against DMSO. (**C**) *Mtb* CFUs following treatment with DCA (5-20 mM) or vehicle control DMSO (0.1% v/v) at 24, 48 or 72 h.p.i. Data represent the median ± range of four donors. CFUs are expressed as percentage of DMSO. Differences were tested by RM Two-way ANOVA with Dunnett's-multiple comparison test against DMSO. Dots represent the mean of triplicates from a single donor. * $p<0.05$, ** $p<0.01$, *** $p<0.001$, **** $p<0.0001$.
